# Lemierre’s Syndrome as a Sexually Transmitted Disease Due to Porphyromonas asaccharolytica Suspected to Be Caused by Pharyngitis Due to Mycoplasma pneumoniae and Epstein-Barr Virus

**DOI:** 10.7759/cureus.28219

**Published:** 2022-08-20

**Authors:** Kota Ueno, Hiroshi Horiuchi, Syusuke Utada, Yoshie Shinomiya, Azusa Sogo, Takao Miyagawa, Shoko Niida, Hiromu Okano, Naoya Suzuki, Tsuyoshi Otsuka, Hiroshi Miyazaki, Ryosuke Furuya

**Affiliations:** 1 Department of Critical Care and Emergency Medicine, National Hospital Organization Yokohama Medical Center, Yokohama, JPN

**Keywords:** sexually transmitted disease, septic thrombophlebitis, mycoplasma pneumoniae infection, lemierre's syndrome, infectious mononucleosis, epstein-barr virus infections

## Abstract

*Porphyromonas asaccharolytica* rarely causes Lemierre's syndrome (LS), which is characterised by sepsis and thrombophlebitis of the internal jugular vein.

An 18-year-old man presented with fever and a sore throat after sexual contact containing oral sex, and his blood sample was positive for atypical lymphocytes. Infectious mononucleosis was suspected initially. However, laboratory data showed elevated D-dimer and procalcitonin levels, and a computed tomography scan showed a thrombus in the internal jugular vein leading to the diagnosis of LS. The *Mycoplasma pneumoniae* (MP) IgM titre was 1:640 (normal, ≦1:40), and the Epstein-Barr nuclear antigen titre (taken 59 days after admission) was 1:10 (normal, <1:10). It was assumed that LS developed after infection with Epstein-Barr virus (EBV) and MP.

LS should be suspected in young patients with fever and sore throat with a history of recent sexual contact. As pharyngitis was considered the cause of LS, evaluation of the preceding infection such as MP or EBV leading to pharyngitis should also be considered.

## Introduction

Lemierre's syndrome (LS) is a disease characterised by sepsis and thrombophlebitis of the internal jugular vein, mostly caused by pharynx infection in healthy young adults mainly with *Fusobacterium* species [1]. Primary pharyngitis, such as *Mycoplasma pneumoniae* infection, is followed by local invasion of the pharyngeal space and internal jugular vein leading to septic thrombophlebitis, with an interval of one to three weeks [2]. Although the source of infection is usually considered to be the oropharynx, bacteria from other parts of the body including the urogenital and intestinal tract are reported to cause LS [3]. Although LS by *Fusobacterium* species suspected to be caused by pharyngeal trauma during oral sex had been reported, LS is rarely recognized as a sexually transmitted disease [4].

Here, we report a case of LS caused by *Porphyromonas asaccharolytica*, which is a bacterium common in the urogenital and intestinal tracts, with suspected previous *Mycoplasma pneumoniae* pharyngitis and infectious mononucleosis (IM) by Epstein-Barr virus (EBV) probably due to sexual contact including oral sex.

## Case presentation

An 18-year-old man was brought to the emergency room complaining of sore throat and neck pain, with a fever of 39°C and chills and tremors. He had sexual contact including oral sex with his new girlfriend a month ago. Since then, he did not have any other contact with others and always wore a mask outside because of the COVID-19 pandemic in Japan. There were no special notes on his medical or family history. 

On admission (day 1), the patient's respiratory rate was 30 breaths/min, SpO_2_ was 95% (nasal cannula, 2 L), blood pressure was 116/64 mmHg, pulse rate was 110 beats/min, and body temperature was 39.4°C. Physical examination showed swelling of the left neck (3 cm), enlarged tonsils, and white moss on the soft palate.

Laboratory results on admission showed elevation of liver enzyme, count of atypical lymphocytes, C-reactive protein, procalcitonin, and D-dimer (Table 1).

**Table 1:** Laboratory results on admission

		normal range
White blood cells (K/mm3)	4.2	3.3–8.6
Segmented neutrophil (%)	62.0	38.0-74.0
Stabbed neutrophil (%)	13.0	0.5-6.5
Lymphocyte (%)	16.0	16.5-49.5
Atypical lymphocyte (%)	+	-
Monocyte	9.0	2.0-10.0
Platelets (K/mm3)	98	150-250
C-reactive protein (mg/dl)	21.23	0.00–0.14
Procalcitonin (mg/dl)	36.4	0.00–0.046
Aspartate aminotransferase (IU/L)	78	13-30
Alanine aminotransferase (IU/L)	79	10-42
Lactate dehydrogenase (IU/L)	302	124-222
D-dimer (mcg/ml)	12.3	< 1.0

**Figure 1:**
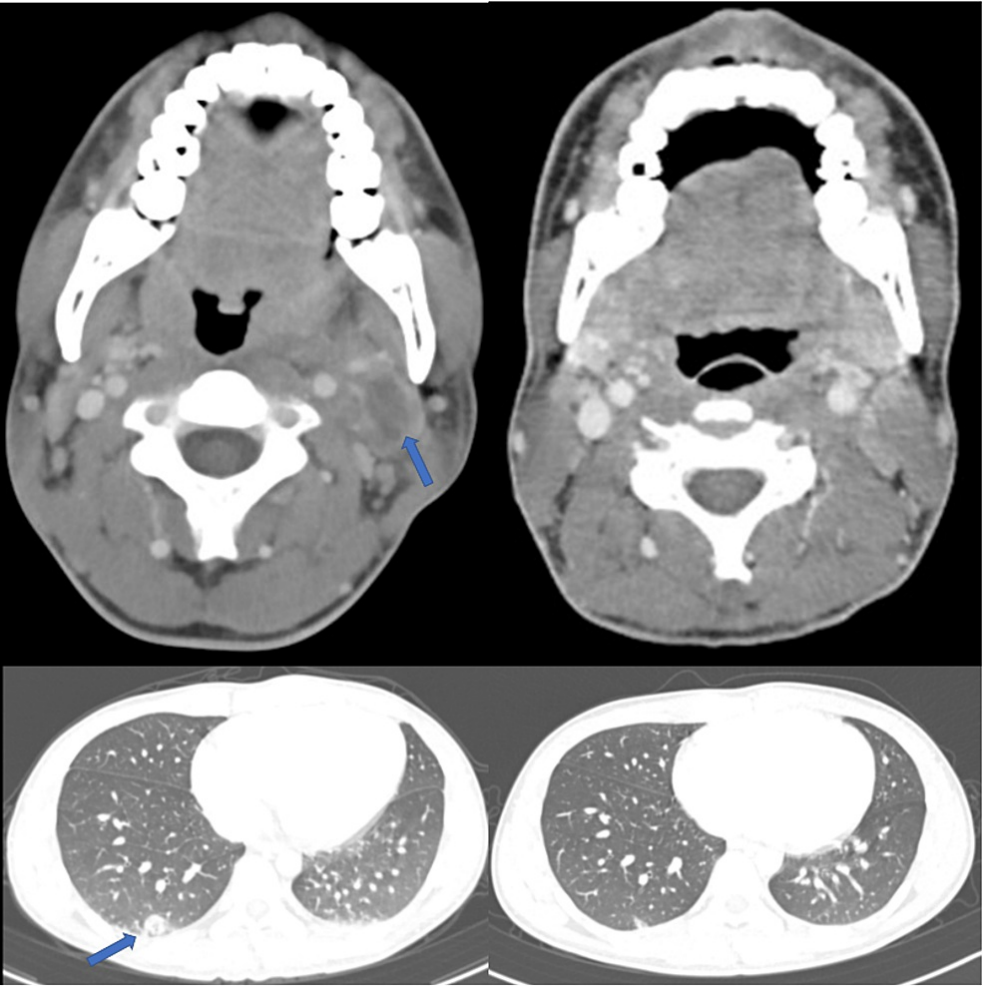
Serial computed tomography (CT) findings Left: Contrast-enhanced computed tomography (CT) of the neck performed on day 1. The upper left image shows a thrombus in the left internal jugular vein. The contrast-enhancing effect suggests abscess formation in the soft tissue around the internal jugular vein (arrows). The image on the lower left shows a round nodule suspected to be a septic embolus (arrows). Right: Contrast-enhanced CT of the cervical region on day 59. The image on the upper right shows the resolution of the neck abscess. The image on the lower right shows the resolution of the pulmonary nodule.

D-dimer was measured for the differentiation of LP. Due to the elevated procalcitonin and D-dimer, contrast-enhanced computed tomography (CT) of the neck, chest, abdomen, and pelvis was performed. The neck CT showed enlarged tonsils and abscess formation around the left internal jugular vein and a thrombus in the left internal jugular vein (Figure 1).

The chest CT showed round nodules suspected to be septic emboli in the upper and lower lobes of the lung. Abdominal CT revealed mild hepatosplenomegaly. The rapid group A streptococcal antigen test results were negative. MP IgM titre (particle agglutination method) was 1:640 (normal, ≦1:40). 

**Figure 2:**
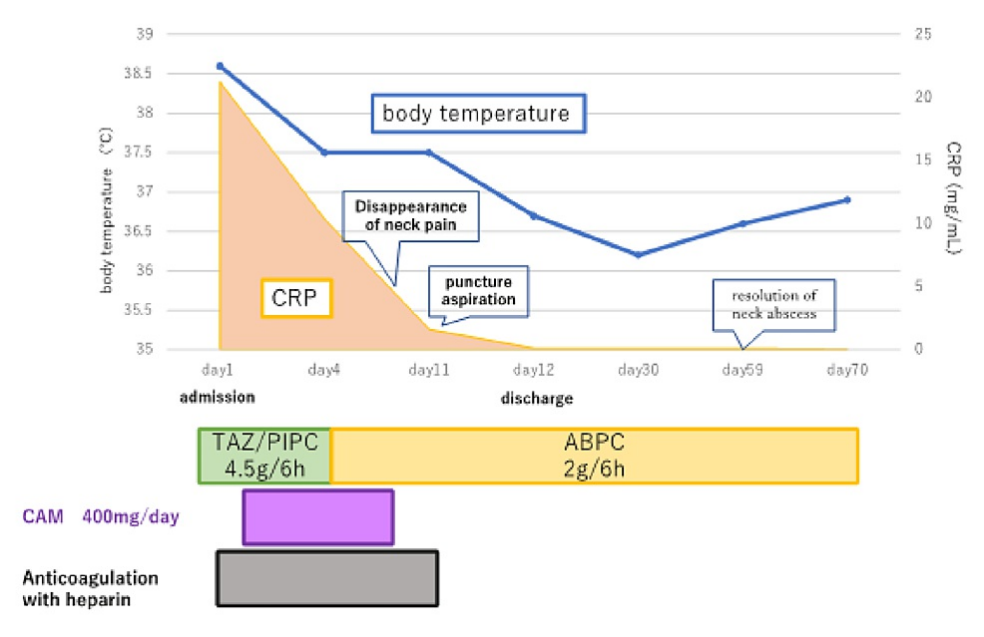
Clinical progress CRP, C-reactive protein; TAZ/PIPC, piperacillin-tazobactam; ABPC, ampicillin; CAM, clarithromycin

Intravenous piperacillin-tazobactam 4.5 g every 6 hours was started for LS, and oral clarithromycin 200 mg every 24 hours was started for MP infection. On day 5, *Porphyromonas asaccharolytica* was detected in the blood culture on admission, and antimicrobial treatment was de-escalated with ABPC 2 g every 6 hours. Anticoagulation with heparin was started for the pulmonary septic emboli from day 1, but was terminated on day 9 considering the risk of haemorrhage following an abscess puncture. On day 11, ultrasound-guided puncture aspiration was performed to treat the abscess. A small amount of fluid was aspirated from the abscess. Bacterial culture of the aspirated fluid was negative. The patient was discharged after two weeks of hospitalisation and continued antimicrobial therapy (Figure 2).

On day 59, the EBV viral capsid antigen (VCA) IgM titre was <1:10 (normal, <1:10), VCA IgG titre was 1:80 (normal, <1:10), early antigen-diffuse and restrict complex IgG titre was <1:10 (normal, <1:10), and EBV-associated nuclear antigen (EBNA) titre was 1:10 (normal, <1:10).

A follow-up CT (day 59) in the outpatient department showed resolution of the internal jugular vein thrombus, neck abscess, and pulmonary nodules (Figure 1). Antimicrobial therapy was completed on day 71 because of the disappearance of the neck abscess on contrast-enhanced CT.

## Discussion

We found two important clinical issues. LS should be suspected in young patients with fever, sore throat, and neck pain after sexual contact including oral sex, and the cause of pharyngitis leading to oropharyngeal mucosal disruption, such as MP and EBV, should be examined. *Porphyromonas asaccharolytica*, which is not a resident bacterium in the oral cavity, was detected as the causative organism; therefore, part of LS can be recognised as a sexually transmitted disease.

First, LS should be suspected in a young patient with fever, sore throat, and neck pain with a recent history of oral sexual contact, who is suspected to have infectious mononucleosis (IM) [1,5]. Such viral or bacterial pharyngitis was reported to be a risk of LS by damaging oropharyngeal mucosa [6]. In a past study, “forcible oral sex” was reported to be a cause of oropharyngeal mucosal disruption [4]. In our case, the patient did not have forceful oral sex with his girlfriend, and it was not physical oropharyngeal mucosal disruption but pharyngitis by MP or EBV transmitted from his girlfriend that was considered a cause of LS.

Cervical swelling in IM is symmetrical, soft, and associated with multiple lymphadenopathy, whereas cervical swelling in LS is mostly unilateral [7, 8]. This is a difference in physical findings. In this case, IM was suspected because of the course of symptoms, sexual contact, and the elevated aminotransferases and atypical lymphocytes in the blood tests. However, the laboratory results showed elevated D-dimer and procalcitonin levels, and we thought it overlapped with LS and EBV infection [9, 10]. In this case, we did not measure the EBV antibody titre on day 1, and we could not confirm the presence of an acute EBV infection. However, the EBNA titre on day 59 suggested that the patient was in a recovery state and that EBV infection might have coexisted with LS.

The incubation period of MP and EBV is reported to be two to three weeks and four to eight weeks respectively [11, 12]. In this case, we suspected MP and EBV were transmitted simultaneously from his girlfriend a month ago, following which pharyngitis was caused by MP leading to LS due to *Porphyromonas asaccharolytica*, which was also suspected to be transmitted and transiently attached in his oral cavity through sexual contact, complicated by EBV-induced IM. A similar case of an 18-year-old woman who developed MP infection followed by LS due to *Fusobacterium nucleatum* complicated by IM due to EBV was reported [9]. Two cases of patients aged 21 years or younger were diagnosed with LS and had coinfection by EBV [13]. When LS is suspected, it is necessary to actively suspect the possibility of prior and multiple infections with MP or EBV.

Secondly, *Porphyromonas asaccharolytica* is a possible causative organism of LS. A meta-analysis reported that *Fusobacterium necrophorum* was detected in 33.2%, *Fusobacterium* species in 5.1%, and *Porphyromonas asaccharolytica* in 0.8% as the pathogen of LS [3]. The meta-analysis also detected non-resident bacteria in the oral cavity, such as *Bacteroides fragilis* and *Spirochete/Treponema* at 1.0% and 0.3%. *Porphyromonas asaccharolytica* is a bacterium common in the urogenital and intestinal tracts [14]. This suggests that part of LS should be recognised as a sexually transmitted disease rather than a simple invasion of oral commensals. In this case, there was a possibility of preceding or simultaneous pharyngitis due to MP and EBV infection, which may have been caused by close oral contact due to sexual contact including oral sex. In addition, it was assumed that they caused pharyngitis and that *Porphyromonas asaccharolytica* was transmitted by oral sex, leading to the mucosal invasion of the bacteria.

The limitation of this study was that we could not evaluate the complication of IM at the time of admission, and the diagnosis of EBV co-infection was obscure.

## Conclusions

In conclusion, LS should be suspected in young patients with a sore throat and neck pain, especially with a recent history of oral sex, and preceding infection that causes pharyngitis leading to oropharyngeal mucosal disruption such as MP and EBV should be examined. In this case, *Porphyromonas asaccharolytica*, a bacterium that does not reside in the oral cavity, caused LS. Therefore, oral sexual contact may trigger LS even without physical oropharyngeal mucosal disruption, and there is a need for a large-scale epidemiological study of complications of other infections or oral sexual contact in the future.
